# A novel surface plasmon resonance sensor development for broflanilide determination using molecularly imprinted polymers and sulphur doped reduced graphene oxide@nickel sulfide nanoparticles

**DOI:** 10.1007/s00604-026-08041-3

**Published:** 2026-04-14

**Authors:** Mustafa Anıl Erbağcı, Kaan Kaplan, Neslihan Özdemir, Hüseyin Enes Altınok, Mehmet Lütfi Yola

**Affiliations:** 1 Department of Nutrition and Dietetics, Faculty of Health Sciences, Hasan Kalyoncu University, Gaziantep, 27010 Türkiye Turkey; 2 Department of Aerospace Engineering, Sivas Science and Technology University, Sivas, 58000 Türkiye Turkey; 3 Department of Machinery and Metal Technologies, Merzifon Vocational School, Amasya University, Amasya, 05300 Türkiye Turkey; 4 Department of Chemical Engineering, Faculty of Engineering, Pamukkale University, Denizli, 20160 Türkiye Turkey; 5 Department of Biology, Faculty of Science, Ankara University, Ankara, 06100 Türkiye Turkey; 6 Integrated Technologies Research Center (BUTAM), Ankara University, Ankara, 06690 Türkiye Turkey

**Keywords:** Broflanilide, Surface plasmon resonance, Nanocomposite, Reduced graphene oxide, Nickel sulfide, Molecularly imprinted polymers

## Abstract

Broflanilide (BRO) is anticipated to become a crucial tool in pest management strategies for combating pests developing resistance to traditional noncompetitive antagonists. This study detailed the development of a novel molecularly imprinted surface plasmon resonance (SPR) sensor for detecting BRO in bottled drinking water and rice samples. The initial step involved the hydrothermal synthesis of a sulphur-doped reduced graphene oxide@nickel sulfide nanoparticles (S-rGO@NiS_2_NPs) nanocomposite. Subsequently, SPR chips incorporating S-rGO@NiS_2_NPs material were fabricated based on the interaction between the gold surface of the SPR chip and the sulphur component within the nanocomposite. Subsequently, a molecularly imprinted SPR sensor based on S-rGO@NiS_2_NPs nanocomposite was developed using UV polymerization, employing methacryloylamidoglutamic acid (MAGA) as the functional monomer and N, N′-azobisisobutyronitrile (AIBN) as the radical initiator. The fabricated sensor demonstrated a linear detection range between 1.0 ng L^− 1^ and 10.0 ng L^− 1^ and achieved a limit of detection (LOD) as low as 0.33 ng L^− 1^. Furthermore, the fabricated SPR sensor was successfully utilized for the analysis of the bottled drinking water and rice samples, producing high recovery. In the final stage, SPR sensor’s stability, selectivity, repeatability, and reproducibility were thoroughly investigated.

**Graphical abstract:**

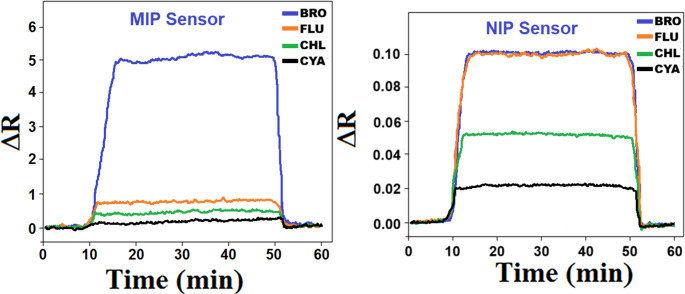

**Supplementary Information:**

The online version contains supplementary material available at 10.1007/s00604-026-08041-3.

## Introduction

Broflanilide (BRO) is a novel meta-diamide insecticide (IRAC Group 30) that acts as an allosteric modulator of insect GABA-gated chloride channels [1–3]. By disrupting chloride ion flux, BRO induces hyperexcitability and death in pests, showing high efficacy even against populations resistant to conventional GABA antagonists [4]. Due to its broad-spectrum activity, BRO is extensively applied to various crops, including cereals, vegetables, and fruits, and is also utilized in public health programs for malaria vector control [5–7]. Despite its effectiveness, the concerns regarding its long-term impact have emerged; the United States Environmental Protection Agency (EPA) has classified BRO as “Likely to be carcinogenic to humans” and in vitro studies suggest potential cytotoxicity and DNA damage [8, 9]. Given its widespread use and the established acceptable daily intake (ADI) of 0.02 mg/kg, the precise monitoring of BRO residues in food and environmental samples is of paramount importance for public health [10].

In literature, chromatographic methods are generally preferred for the determination of BRO and its metabolites (DM-8007 and S(PFP-OH)−8007) in agricultural products and soil. The most employed approach involves modifications of the QuEChERS (Quick, Easy, Cheap, Effective, Rugged, and Safe) method for sample preparation. For quantitative analysis and confirmation, liquid chromatography tandem mass spectrometry (LC-MS/MS) and ultra high performance liquid chromatography quadrupole time of flight mass spectrometry (UHPLC-QTOF-MS) provide high sensitivity and reliability [5, 11]. These techniques enable the detection of trace residues of BRO and its metabolites at very low concentrations with a limit of quantification (LOQ) typically around 0.001–0.005 mg kg^− 1^ [5]. These analytical techniques entail substantial practical constraints, as they are generally inefficient in terms of time, economically burdensome, and dependent on extensive instrumental resources. In particular, the development of analytical methods with rapid, selective, and instantaneous measurement responses is necessary for the early diagnosis of metabolic disorders resulting from pesticide exposure. Among analytical methods, SPR sensors are distinguished by their rapid response times and their compatibility with microfluidic systems, enabling the development of integrated “lab-on-a-chip” platforms that combine sample handling, chemical analysis, and data processing within a single device [12]. Additionally, SPR sensors allow real time and simultaneous evaluation of molecular association and dissociation processes [13]. Owing to these advantages, SPR based sensor systems have been widely applied in food analysis, including the detection of toxins and nutritional additives [14]. Especially, nanomaterial and molecularly imprinted polymers (MIPs) based SPR sensor applications have been frequently used in recent years [15].

Among nanomaterials, graphene oxide (GO) is a important derivative of graphene, recognized for its exceptional optical and mechanical attributes. The chemical modification of GO has often involved the use of hydrazine hydrate or hydrazine. This process can cause the formation of rGO, which is capable of yielding graphene sheets [16]. The surface of rGO sheets typically contains various functional groups, such as epoxy, hydroxyl, carboxyl, and carbonyl. To enhance the chemical activity and electrical properties of these graphene derivatives, several approaches have been explored [17]. Among these, the dopping has emerged as a beneficial technique for boosting the catalytic efficiency [18]. Dopping involves the incorporation of impurities into pure semiconductors to alter their chemical characteristics. Similarly, the chemical functionalization of graphene nanomatarials with heteroatoms is widely recognized as a potent strategy for enhancing their electrical and mechanical attributes. Hence, the electrical conductivity of rGO sheet was enhanced through the incorporation of sulphur dopping [19]. NiS_2_ has been utilized as both a synergistic agent and a cocatalyst alongside semiconductive materials to increase catalytic effectiveness [20]. rGO functions as an efficient electron scavenger in NiS_2_-based materials, gathering photoinduced electrons and channeling them towards the surface of nanocomposite.

Molecularly imprinting is an advanced polymer fabrication strategy typically implemented through a three-stage process. Initially, functional monomers interact with the target molecule via covalent and/or non-covalent interactions, leading to the formation of a pre polymerization complex [21]. This complex is subsequently subjected to polymerization, and the template molecule is then removed using an appropriate solvent, resulting in the formation of selective polymeric matrices capable of specifically recognizing the original template. MIPs provides several advantages, including straightforward synthesis, cost-effectiveness, high selectivity and sensitivity, and notable physicochemical stability. Owing to these properties, MIPs are extensively utilized in contemporary food safety analysis and monitoring applications [22].

This study presented a cutting-edge methodology for the precise detection of BRO insecticide, including MIPs in conjunction with a sulphur-doped reduced graphene oxide@nickel sulfide nanoparticles. The core of this research involved in the synthesis of the sulphur-doped reduced graphene oxide@nickel sulfide nanoparticles through a hydrothermal process. Subsequently, this nanocomposite was integrated into a SPR chip and decorated by a coating of MIPs. This advanced SPR sensor revealed the first application in quantifying BRO amounts in the bottled drinking water and rice samples on existing literature.

## Materials and methods

### Materials and instrumentation

Flubendiamide (FLU), chlorantraniliprole (CHL), cyantraniliprole (CYA), sodium sulfide (Na_2_S), ethylenediaminetetraacetic acid (EDTA), nickel(II) sulfate hexahydrate (NiSO_4_⋅6H_2_O), sodium thiosulfate (Na_2_S_2_O_3_⋅H_2_O), MAGA, ethylene glycol dimethacrylate (EGDMA), 2-hydroxyethylmethacrylate (HEMA), AIBN, phosphate buffer (0.1 mol L^− 1^, PB) and sodium chloride (NaCl) were purchased by Sigma-Aldrich (USA). The apparatus for analytical and structural analysis was given in Supplementary Data.

### Preparation of rGO and S-rGO

**rGO** was prepared according to the protocol belonging to our previous paper [23]. For S-rGO synthesis, pure water (10.0 mL) was combined with rGO suspension (5.0 mL, 4.00 mg mL^− 1^). After that, 1.0 mol L^− 1^ Na_2_S (5.0 mL) was transferred into the mixture, which was then subjected to sonication for 1 h, providing sulphur-doped rGO flakes (sonication parameters were operating frequency of 20 kHz and power of 60.0% amplitude). The obtained product underwent multiple washing steps with pure water to remove any contaminants and dried at 25 °C. The final product was tagged as **S-rGO**.

### Preparation of NiS_2_NPs and S-rGO@NiS_2_NPs nanocomposite

The quantities of 0.10 mol L^− 1^ NiSO_4_·6H_2_O, 0.10 mol L^− 1^ EDTA, and 0.10 mol L^− 1^ Na_2_S_2_O_3_·H_2_O were measured and introduced into a 100 mL flask. The mixture was then subjected to the magnetic agitation to provide homogeneous dispersion throughout. The prepared solution was transferred into a 100 mL Teflon-lined autoclave, where the solution was heated to 100 °C at the heating rate of 5 °C min^− 1^ for 30 h. After this hydrothermal treatment including the rotation speed of 500 rpm and the magnetic stirring of 15 min, the solution was allowed to cool. The resulting products were subsequently isolated by centrifugation and washed three times with pure water. Finally, the purified nanomaterials were dried at 100 °C for 10 h, producing NiS_2_NPs [24].

For the synthesis of S-rGO@NiS_2_NPs nanocomposite, after 50.0 mL of pure water was combined with 4.0 mg mL^− 1^ rGO (10.0 mL), 0.10 mol L^− 1^ NiSO_4_·6H_2_O (2.0 mL), 0.10 mol L^− 1^ EDTA (2.0 mL), and 0.10 mol L^− 1^ Na_2_S_2_O_3_·H₂O (4.0 mL) were added to rGO suspension (10.0 mL). Then, the entire mixture was sonicated for 1 h (sonication parameters were operating frequency of 20 kHz and power of 60.0% amplitude) and this mixture was transferred into a 100 mL Teflon-lined autoclave. After sealing, the autoclave was maintained at 100 °C at the heating rate of 5 °C min^− 1^ for 40 h to facilitate the hydrothermal reaction based on the rotation speed of 500 rpm and the magnetic stirring of 15 min. Finally, the obtained product was washed three times with pure water (**S-rGO@NiS**_**2**_**NPs**) [19].

### SPR chip modification with S-rGO@NiS_2_NPs nanocomposite and the preparation of MIP film modified S-rGO@NiS_2_NPs-functionalized SPR chip

Initially, SPR chips were cleaned by submerging them in a conical flask for 20 min with 10.0 mL of an acidic piranha solution (3:1 H_2_SO_4_:H_2_O_2_, v/v). Then, the SPR chips were dried under an inert argon atmosphere to preserve their pristine condition. To enable a strong gold-sulphur interaction, S-rGO@NiS_2_NPs nanocomposite suspension (1.0 mL, 20.0 mg mL^− 1^) was carefully applied to the gold surface of SPR chip. These modified chips (**S-rGO@NiS**_**2**_**NPs/SPR**) were subsequently stored under an argon atmosphere to maintain their integrity and readiness for further use. When the interaction between the gold surface of SPR chip and the sulphur component in S-rGO@NiS_2_NPs nanocomposite was investigated, the sulphur atom formed a gold–thiolate bond (Au–S) with SPR surface gold atoms. This interaction was classified as chemisorption, often described as covalent or covalent-like bonding [25]. Typical Au–S bond energy also was 160–210 kj mol^− 1^ and this was strong enough to form stable self-assembled monolayers (SAMs) used in SPR biosensors [26].

For the fabrication process of MIP film modified S-rGO@NiS_2_NPs-functionalized SPR chip, a MAGA (100.0 mmol L^− 1^)-BRO (50.0 mmol L^− 1^) complex solution was initially prepared by combining the components in a 2:1 molar ratio within 6.0 mL of phosphate buffer at pH 6.0. We can explain specific reasons for choosing MAGA monomer as follows: (i) Methacrylate-based polymers are known for their good mechanical stability and chemical resistance, which are essential for sensor robustness and MAGA can be polymerized using common initiators (e.g., AIBN) offering flexibility in MIPs synthesis [27]. (ii) The carboxylic acid and amide groups in the structure are hydrophilic, improving the mass transfer [28]. This MAGA-BRO pre-complex occured in two ways. (i) BRO’s amide group (–CONH–) interacted with MAGA carboxyl group (–COOH/–COO⁻), donating BRO’s H-bond to the oxygen of MAGA carboxylate [BRO (–NH) ⋯ O = C–O− (MAGA)]. (ii) Amide carbonyl (C = O) group of BRO interacted with MAGA amino group (–NH_2_/–NH_3_^+^) and the carbonyl oxygen of BRO acted as a hydrogen bond acceptor [MAGA (NH_3_^+^​) ⋯ O = C (BRO)]. Subsequently, the complex solution (3.0 mL) was mixed with 1.0 mg of AIBN, 6.0 mL of HEMA (0.10 mol L^− 1^), and 6.0 mL of EGDMA (0.30 mol L^− 1^) to form a polymerization mixture. This mixture (1.0 mL) was then applied onto S-rGO@NiS_2_NPs/SPR chip using a spin coating technique with a rotation speed of 5000 rpm for coating time of 30 s, facilitating the formation of a homogeneous, single-layered polymeric structure. To complete the imprinting process, UV polymerization was carried out on SPR chip for 30 min, yielding MIP film modified S-rGO@NiS_2_NPs-functionalized SPR chip (**MIP/S-rGO@NiS**_**2**_**NPs/SPR)**. Mercury vapor lamp with wavelength range from 315 to 400 nm, emitting a broad spectrum of UV light, was used as a light source and its intensity can range from 10 to 100 mW cm^− 2^. For comparative analysis, a NIP film modified S-rGO@NiS_2_NPs-functionalized SPR chip (**NIP/S-rGO@NiS**_**2**_**NPs/SPR**) was also developed using an identical procedure with omitting BRO target molecule.

### BRO removal from MIP/S-rGO@NiS_2_ NPs/SPR and analysis process

To effectively eliminate electrostatic and hydrogen bonding interactions between MAGA monomer and BRO analyte, NaCl solution (0.1 mol L^− 1^, 10.0 mL) was used for the removal of BRO molecules. MIP/S-rGO@NiS_2_NPs/SPR chip was immersed in NaCl solution for 10 min to complete the desorption of BRO. After that, MIP/S-rGO@NiS_2_NPs/SPR chip with free of BRO molecules was subjected to the vacuum-drying at 25 °C, providing its regeneration and readiness for subsequent usage.

Upon integrating MIP/S-rGO@NiS_2_NPs/SPR chip with the removed BRO molecules into SPR cell, the system was initially brought to equilibrium by flushing 3.0 mL of PB (pH 6.0) for 10 min at a flow rate of 1.0 mL min^− 1^. Subsequently, the adsorption phase involving 1.0 mL aliquots of BRO solutions with concentrations spanning from 1.0 to 10.0 ng L^− 1^ was performed. Each adsorption solution was interacted with MIP/S-rGO@NiS_2_NPs/SPR chip for a duration of 40 min. This was then followed by the desorption phase, which was provided to apply 3.0 mL of 0.1 mol L^− 1^ NaCl solution over a 10-min period. Thus, a complete “adsorption–desorption–regeneration” cycle was executed for each BRO concentration.

### Sample preparations

For the analysis of the bottled drinking water, the sample (10.0 mL) was initially transferred into a 25.0 mL conical flask. This sample then underwent centrifugation with a rotation speed of 1000 rpm for 5 min to eliminate any solid residues. Then, the resulting clear solution was diluted with 0.1 mol L^− 1^ PB at pH 6.0. This dilution was carried out to ensure that BRO analyte concentration fell within the linear detection range of the SPR system. Finally, the prepared water sample was introduced into the SPR cell for subsequent analysis.

Secondly, a 1.00 g rice sample, sourced from a local market, initiated the process with a wash using 10.00 mL of distilled water to eliminate any surface contaminants. Following drying treatment, the rice was pulverized into a powder, then introduced to a 10.00 mL solution including in ethyl alcohol and acetonitrile in a 1:1 (v/v) ratio. This mixture was subjected into homogenization for 10 min, then the centrifugation for 10 min at a rotation speed of 1000 rpm was completed. The resulting clear supernatant was subsequently diluted with 0.1 mol L^− 1^ PB at pH 6.0 to ensure its concentration fell within the calibration linearity limits. Ultimately, a successful BRO analysis in rice sample was conducted utilizing tMIP/S-rGO@NiS_2_NPs/SPR chip.

## Results and discussion

### Structural analysis of S-rGO@NiS_2_NPs nanocomposite

**Fig. 1:**
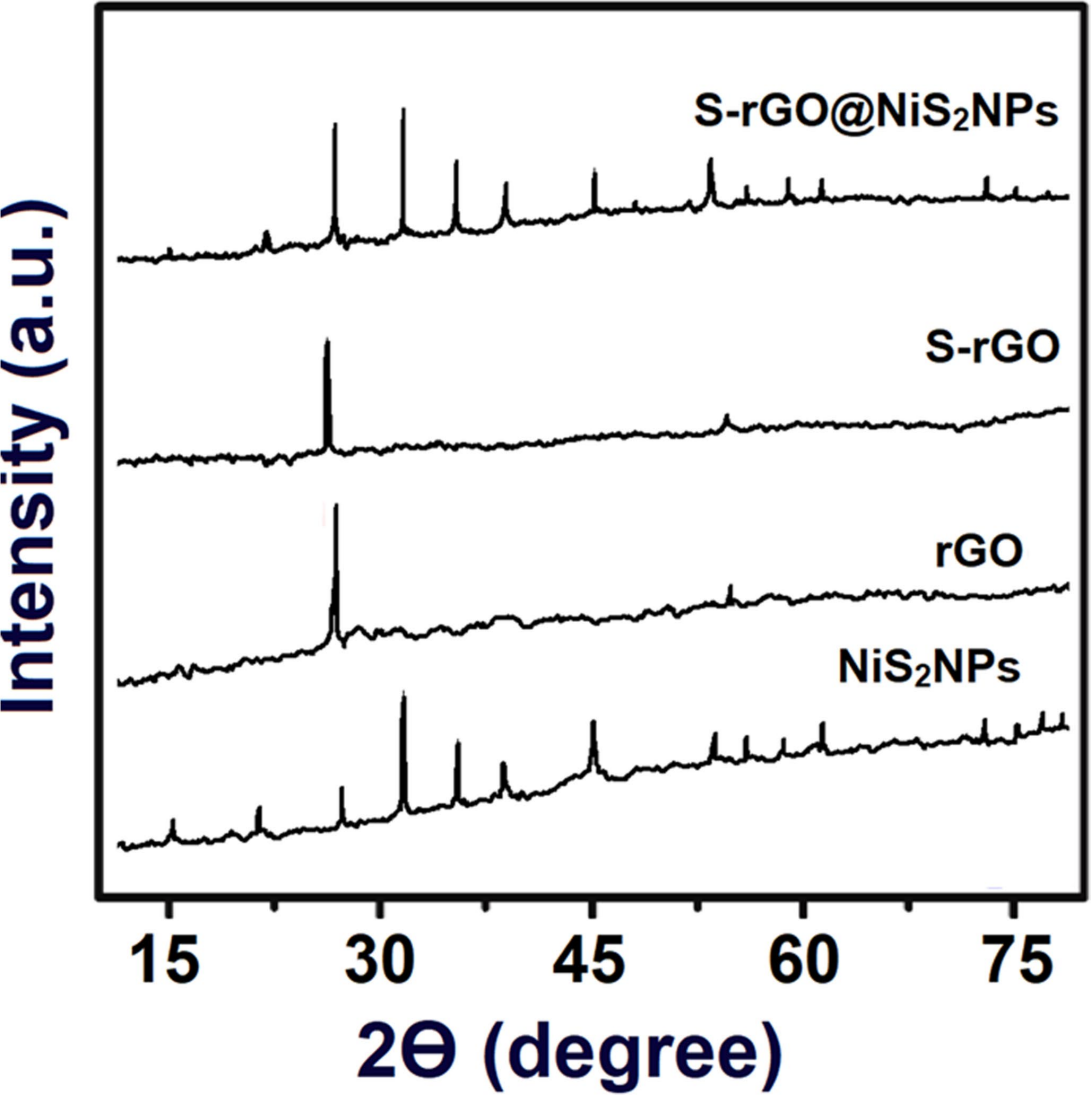
XRD pattern of rGO, S-rGO, NiS_2_NPs, and S-rGO@NiS_2_NPs nanocomposite

XRD analysis was employed to confirm the structural characteristics and phase composition of the synthesized nanomaterials. XRD profiles for rGO, S-rGO, NiS_2_NPs, and S-rGO@NiS_2_NPs depicted distinct features. The sharp and narrow peaks for the nanomaterials obviously suggested their crystalline nature. The rGO’s XRD spectrum produced smooth patterns with prominent peaks, which was corresponded to the absence of residual functional groups on the rGO sheets. A strong and sharp peak at approximately 2θ = 27.06° (Fig. 1) verified the presence of rGO. Similarly, XRD results for S-rGO demonstrated a peak at around 2θ = 27.06°, indicating its presence. Notably, a comparison of XRD patterns for rGO and S-rGO revealed no substantial changes in their characteristic peaks, apart from a slight downward shift of the (002) peak position for rGO when incorporated into S-rGO. Moreover, the specific diffraction peaks for NiS_2_NPs were seen corresponding to (001), (110), (111), (200), (012), (112), (202), (122), (130), (113), (222), (320), (231), (004), (223), (114), (133), (024), and (241) planes [19]. According to XRD spectrum of S-rGO@NiS_2_NPs nanocomposite, the peaks corresponding to (001), (110), (111), (200), (012), (112), (202), (122), (130), (113), (222), (320), (231), (004), (223), (114), (133), (024), and (241) planes verified the presence of NiS_2_NPs, while a less prominent peak at (002) indicated the presence of S-rGO, indicating a successful incorporation of NiS_2_ with S-rGO [19]. Lastly, crystallite size was calculated by Scherrer’s equation, and the crystal sizes were found to be 6.19 nm for rGO, 11.69 nm for S-rGO, 16.37 nm for NiS_2_NPs and 21.89 nm for S-rGO@NiS_2_NPs nanocomposite. These results indicated the uniform synergistic interaction between NiS_2_NPs and S-rGO.

**Fig. 2:**
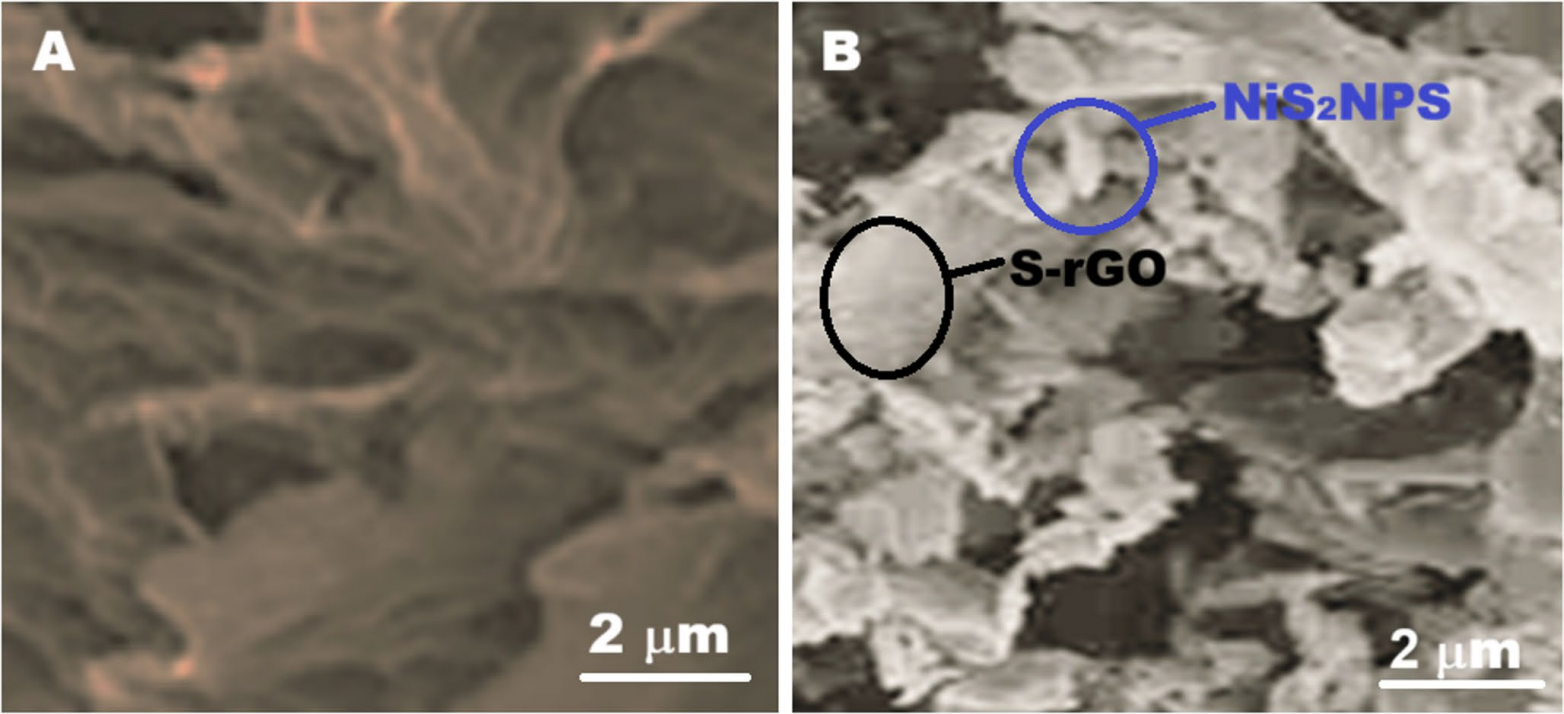
SEM images of (**A**) rGO and (**B**) S-rGO@NiS_2_NPs nanocomposite

SEM images (Fig. 2) suggested some insights into the morphologies of both pristine rGO and S-rGO@NiS_2_NPs nanocomposite. Figure 2A obviously demonstrated that rGO possessed a flexible, nanosheet-like architecture [29]. As depicted in Fig. 2B, the resulting S-rGO@NiS_2_NPs nanocomposite showed a distinctive morphology in the terms of granular and flake-like formations, confirming the noticeable agglomeration of NiS_2_NPs on the surface of S-rGO [19].

Energy dispersive x-ray (EDX) was conducted on S-rGO@NiS_2_NPs nanocomposite to evaluate the distribution of its elements (Fig. S1A). The resulting EDX spectrum showed clear, and sharp peaks attributing to carbon, nickel, sulphur, and oxygen. The presence of distinct peaks for each element confirmed their homogeneous dispersion through the nanocomposite. Fig. S1B demonstrated N_2_ adsorption isotherms of rGO, S-rGO, NiS_2_NPs, and S-rGO@NiS_2_NPs nanocomposite and rGO, S-rGO, NiS_2_NPs, and S-rGO@NiS_2_NPs nanocomposite as the mesoporous materials depicted IV types of N_2_ adsorption curves. BET surface areas depicted distinct values including 11.19 m^2^ g^− 1^ for rGO, 23.07 m^2^ g^− 1^ for S-rGO, 31.37 m^2^ g^− 1^ for NiS_2_NPs and 72.67 m^2^ g^− 1^ for S-rGO@NiS_2_NPs nanocomposite. This important increase in BET surface area in S-rGO@NiS_2_NPs nanocomposite in comparison with S-rGO and NiS_2_NPs constituents was owing to the synergistic interaction of its multiple components. This interaction could facilitate the additional catalytic active sites within S-rGO@NiS_2_NPs nanocomposite.

FTIR spectra of rGO, S-rGO, NiS_2_NPs, and S-rGO@NiS_2_NPs nanocomposite were also observed on Fig. S2. FTIR spectrum of rGO showed the presence of several functional groups including carbonyl-carboxyl (CO), carbonyl (C = O), hydroxyl (O-H), and aromatic (C = C). Specifically, a distinct peak at 3712 cm^− 1^ was attributed to O-H group and C-H group was identified by a broad band ranging from 2835 to 2990 cm^− 1^. An absorption peak at 1650 cm^− 1^ suggested the presence of C = C bond, while C = O group was responsible for the peak at 1750 cm^− 1^. Moreover, a peak at 1225 cm^− 1^ was corresponded to C-O stretching of epoxy groups, and a peak at 1029 cm^− 1^ was attributed to the broadening of C-O bond in alkoxy groups [29]. FTIR analysis for S-rGO revealed additional characteristic peaks including the expanding pulsation of O-H group at 3440 cm^− 1^ and the presence of –CH_2_- group at 2945 cm^− 1^ [30, 31]. In addition, the peaks at 1661 cm^− 1^ and 1572 cm^− 1^ were attributed to the aromatic sp^2^-hybridized C = C bonds and the extending pulsation of the C-OH bond was related to a peak at 1235 cm^− 1^, while C-O bond’s extending pulsation was corresponded to a peak at 1112 cm^− 1^. Additionally, O-H group’s stretching vibration was associated with a peak at 3442 cm^− 1^. Furthermore, the absorption peaks at 2933 cm^− 1^ and 1631/1548 cm^− 1^ were attributed to –CH_2_- group and aromatic sp^2^-hybridized C = C bonds, respectively. Lastly, a peak at 1052 cm^− 1^ was corresponded to S = O bond, confirming the successful integration of sulphur atoms into the rGO structure. FTIR spectrum of NiS_2_NPs revealed several specific absorption bands. Absorption peak at 630 cm^− 1^ was attributed to the twisting vibration within the Ni-S-Ni framework, indicating the extending frequencies of various atomic bonds within the material. The presence of a hydroxyl group was verified by a peak at 3691 cm^− 1^. Furthermore, the distinct peaks at 1063 and 1251 cm^− 1^ corresponded to the -C-O (alkoxy) and -C-O (epoxy) groups, respectively. The Ni-S bond was also demonstrated by a peak at 922 cm^− 1^ on FTIR spectrum of S-rGO@NiS_2_NPs nanocomposite. Additionally, a small absorption peak at 827 cm^− 1^ was observed, which was related to C-S vibrations [19].

The high-resolution C1s peaks for both rGO and S-rGO were presented in Fig. S3A and Fig. S3B, respectively. The notable changes upon sulphur dopping were a decrease in the C-O bond peak and an increase in the C-S bond peak, indicating that sulphur atoms were substituted oxygen functionalities within the rGO structure [32]. The analysis of the high-resolution S2p peak in S-rGO revealed its deconvolution into two prominent peaks at 164.11 eV and 165.23 eV, which were characteristic of thiophene-type sulphur. In addition, a less intense peak at 169.03 eV was indicative of oxidized-type sulphur. Thus, these specific peak positions and their relative intensities served as an evidence of successful sulphur dopping within the rGO structure (Fig. S3C) [33]. Moreover, S-rGO displayed a relative sulphur concentration of 4.0%. This finding suggested that the regulating the content of incorporated sulphur by adjusting the sulfur-to-carbon ratio during the synthesis process was is a little difficult.

### FTIR and AFM analysis of MIP film modified S-rGO@NiS_2_NPs-functionalized SPR chip

**Fig. 3:**
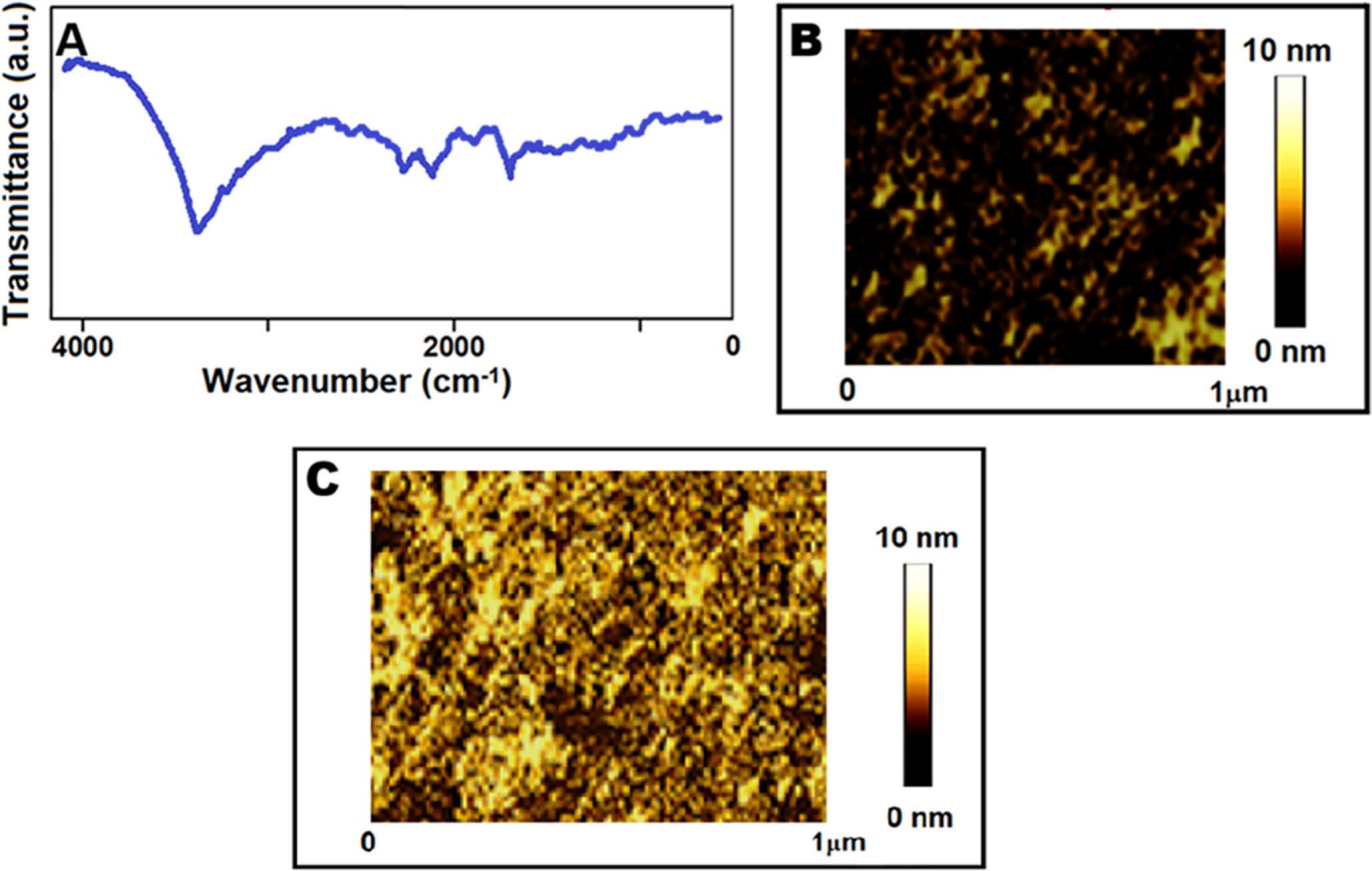
(**A**) FTIR spectra of MIP film modified S-rGO@NiS_2_NPs-functionalized SPR chip with BRO removal; AFM images of (**B**) bare SPR chip and (**C**) MIP film modified S-rGO@NiS_2_NPs-functionalized SPR chip with BRO removal

Following the removal of BRO, FTIR analysis including S-rGO@NiS_2_NPs/SPR provided obvious evidence of characteristic absorption peaks (Fig. 3A). Specifically, the bands at 3601 cm^− 1^ and 3022 cm^− 1^ were attributed to the hydroxyl stretching vibrations of MAGA and HEMA, respectively. The stretching of –CH bonds was related to a band at 1761 cm^− 1^, while a peak at 1447 cm^− 1^ corresponded to the carboxyl-carbonyl stretching and –COO– stretching. These consistent spectral peaks confirmed the successful fabrication of MIP film modified S-rGO@NiS_2_NPs-functionalized SPR chip.

AFM images (Fig. 3B and C) clearly depicted the surface morphology dissimilarities between a bare SPR chip and MIP film modified S-rGO@NiS_2_NPs-functionalized SPR chip. The average surface roughness of 4.03 ± 0.02 nm for bare SPR chip and 46.12 ± 0.03 nm for MIP film modified S-rGO@NiS_2_NPs-functionalized SPR chip were calculated, verifying the successful formation of BRO-imprinted polymeric layers on SPR chip surface. Ellipsometry measurements were also conducted and the resulting thickness of MIP film modified S-rGO@NiS_2_NPs-functionalized SPR surface was determined to be 43.43 ± 0.12 nm, as illustrated in Fig. S4. This consistency across measurement methods supported the successful formation of a homogeneous and monolayer film.

### pH effect on MIP film modified S-rGO@NiS_2_NPs-functionalized SPR chip and the modification effect on SPR signals

**Fig. 4:**
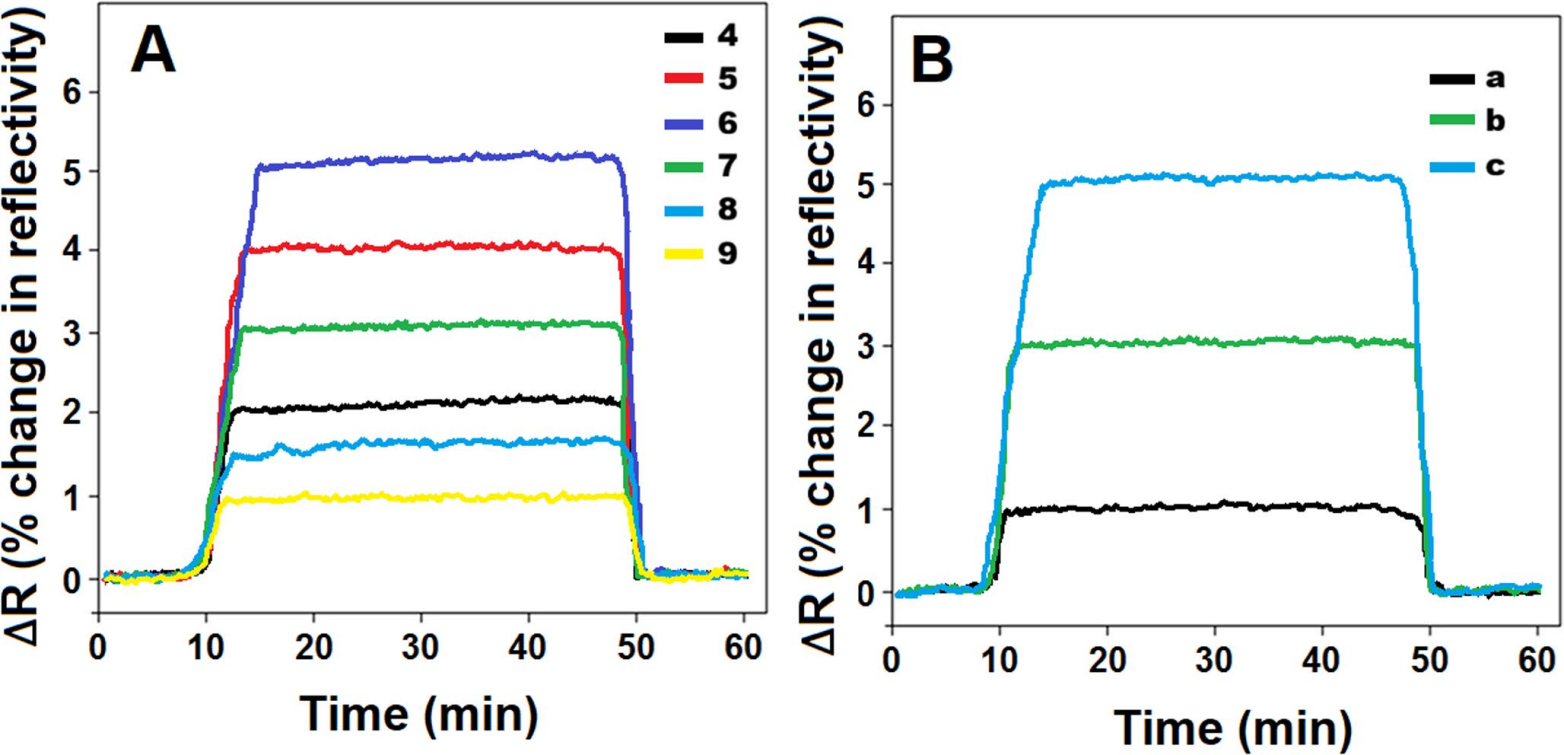
(**A**) SPR sensorgrams for 10.0 ng L^− 1^ BRO in different pHs of PB and (**B**) SPR sensorgrams on MIP/SPR (curve a), MIP/S-rGO/SPR (curve b), and MIP/S-rGO@NiS_2_NPs/SPR (curve c) in presence of 10.0 ng L^− 1^ BRO

The interaction between acidic MAGA monomer and BRO analyte was significantly influenced by pH. In an acidic medium, MAGA monomer showed minimal ionization, which consequently resulted in a reduced interaction with BRO. Nonetheless, as the pH increased, MAGA monomer became more ionized, thus, providing its interaction with BRO analyte. Conversely, under basic conditions (pH > 7.0), the monomer-BRO interaction was substantially diminished, resulting from the simultaneous ionization of both MAGA monomer and BRO analyte. These electrostatic repulsion forces consequently decreased the affinity on SPR chip surface. Thus, the most effective interaction between the monomer and the analyte was observed in a slightly acidic medium, specifically at a pH of 6.0 (Fig. 4A) [34].

Figure 4B demonstrated the impact of surface modification on SPR signals in the existence of 10.0 ng L^− 1^ BRO. When the interaction between BRO analyte and sensor was assessed using MIP/SPR, only a minimal SPR sensor response was recorded (curve a), indicating a limited initial interaction. Conversely, a substantial increase in SPR sensor signal was seen when SPR chip underwent modification with S-rGO (curve b). This notable increase corresponded to two primary factors: (i) the formation of self-assembled monolayers resulting from strong sulphur-gold covalent bonds [15], and (ii) the high catalytic activity endowed to rGO by sulfur doping [35, 36]. Lastly, the highest SPR signal was observed using MIP/S-rGO@NiS_2_NPs/SPR (curve c) because NiS_2_NPs was used as a synergetic agent and large surface areas for the catalytic performance [20].

### Optimization

#### Mole ratio MAGA monomer to BRO effect

In studies involving molecularly imprinted sensors, the stoichiometric ratio for the pre-complex formation between the target molecule and the monomer is significant. For instance, an insufficient amount of MAGA might diminish the specific MAGA-BRO interaction, consequently leading to fewer BRO-specific polymeric cavities on SPR chip. Conversely, an excessive MAGA concentration provided non-specific interactions between the monomer and the nanomaterial on SPR chip. To optimize sensor performance, a pre-complex was thus prepared using 100.0 mmol L^− 1^ MAGA monomer and 50.0 mmol L^− 1^ BRO, yielding the most significant SPR signal, as detailed in Fig. S5A.

#### Desorption time effect

The effective removal of BRO molecule from MIP/S-rGO@NiS_2_NPs/SPR chip was correlated with the enhanced sensor sensitivity. A significant increase in SPR signals was observed up to 10 min desorption time during the desorption process. The subsequent stabilization of these SPR signals after 10 min indicated the complete removal of BRO molecules from the SPR chip surface (Fig. S5B).

#### Sensitivity of MIP film modified S-rGO@NiS_2_NPs-functionalized SPR chip

**Fig. 5:**
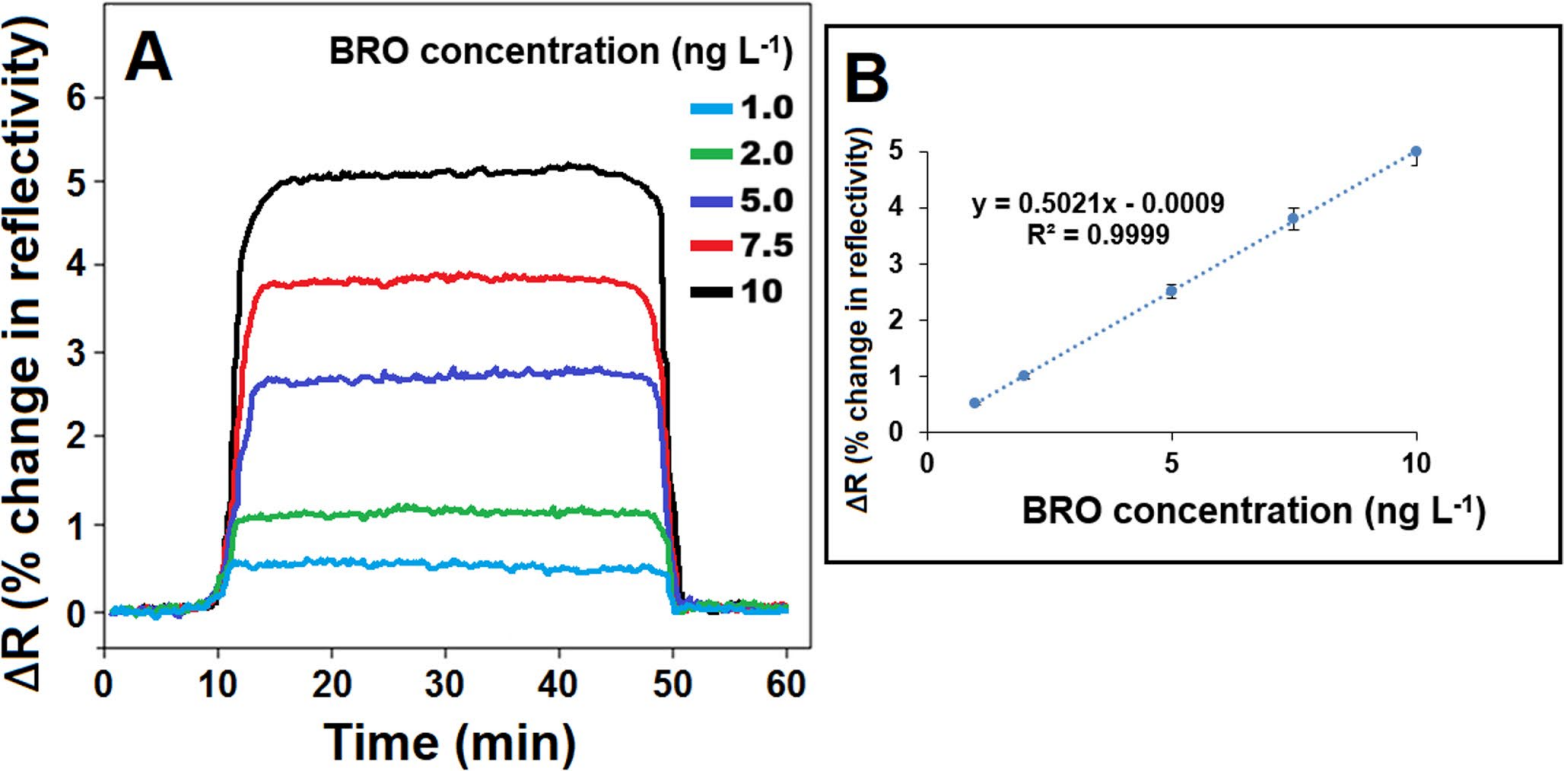
(**A**) SPR sensorgrams with different BRO concentration on MIP/S-rGO@NiS_2_NPs/SPR chip in existence of pH 6.0 of PB (from 1.0 to 10.0 ng L^− 1^ BRO) and (**B**) Calibration curve of BRO concentrations against SPR signals

The analytical performance of MIP/S-rGO@NiS_2_NPs/SPR chip was demonstrated using SPR sensorgrams (Fig. 5A), which obtained SPR responses across BRO concentration range from 1.0 to 10.0 ng L^− 1^. A clear linear correlation was observed between SPR sensor signals and the BRO concentration, producing the calibration equation of y(ΔR) = 0.5021x(C_BRO_, ng L^− 1^)−0.0009 (Fig. 5B). The MIP/S-rGO@NiS_2_NPs/SPR sensor showed exceptional sensitivity, achieving a LOQ of 1.0 ng L^− 1^ and a LOD of 0.33 ng L^− 1^, thus verifying its strong analytical performance (the related equations provided in the supplementary data). A review of the scientific literature revealed that LC-MS/MS and UHPLC analytical techniques were developed for BRO detection [11, 37, 38]. Nonetheless, these techniques were not considered environmentally friendly because they needed the intensive usage of chemical agents. The long analysis times and the need for specialized analysts were other disadvantages. In addition, LC-MS/MS was strictly limited to laboratory settings due to its very high cost and operational complexity. It required extensive sample cleanup (e.g., solid-phase extraction, liquid-liquid extraction) to remove interferences. The sample preparation in UHPLC methods could be simpler than LC-MS/MS, but often required filtration, dilution, or basic extraction steps. LC-MS/MS and UHPLC systems were lab instruments, bulky, and required relatively stable conditions, thus their portability was low. However, SPR systems were designed for point-of-care or field applications. SPR exceled in real-time, label-free interaction analysis with relatively less sample preparation and increasing potential for on-site applications. In this study, a hydrothermal treatment was especially utilized for the synthesis of the S-rGO@NiS_2_NPs nanocomposite, resulting in minimal waste generation and high-yield production. Hence, we can say that the MIP/S-rGO@NiS_2_NPs/SPR system offers a rapid and robust detection method for significant metabolic disorders that arise from exposure to important insecticides.

#### Recovery

To validate MIP/S-rGO@NiS_2_NPs/SPR chip, some recovery measurements were conducted using the bottled drinking water and rice samples. The results (Table 1) depicted nearly 100.00% recovery, verifying the sensor’s high accuracy and selectivity in potential matrix effects. For comparison, BRO detection in the bottled drinking water and rice samples was performed by a valid analytical technique in the literature such as LC-MS/MS [37]. As shown in Table 1, no important difference was found between the obtained results using the MIP/S-rGO@NiS_2_NPs/SPR chip and LC-MS/MS methods. The standard addition method was also applied to the real bottled drinking water and rice samples for BRO analysis, yielding the calibration equation of y(ΔR) = 0.5088x(C_BRO_, ng L^− 1^)−0.1037 for real bottled drinking water sample and the calibration equation of y(ΔR) = 0.5059x(C_BRO_, ng L^− 1^)−0.1012 for rice sample. The notable harmony between the slopes of both the direct calibration and standard addition calibration equations strongly suggested the high selectivity of the proposed MIP/S-rGO@NiS_2_NPs/SPR chip in presence of other matrix effects (e.g., humic acid, inorganic salts, other trace pesticides).

**Table 1:** Recovery results of BRO (*n* = 6)

MIP/S-rGO@NiS_2_NPs/SPR				LC-MS/MS	
Sample	Added BRO (ng L^− 1^)	Found BRO (ng L^− 1^)	*Recovery (%)	Found BRO (ng L^− 1^)	*Recovery (%)
Bottled drinking water	-	2.02 ± 0.03	-	2.03 ± 0.02	-
	2.00	4.03 ± 0.03	100.25 ± 0.02 *RSD = 0.049	4.04 ± 0.03	100.25 ± 0.02 *RSD = 0.049
	5.00	7.01 ± 0.04	99.86 ± 0.06 *RSD = 0.147	7.02 ± 0.04	99.86 ± 0.02 *RSD = 0.049
	7.00	9.03 ± 0.01	100.11 ± 0.04 *RSD = 0.098	9.04 ± 0.05	100.11 ± 0.03 *RSD = 0.073
Rice	-	1.93 ± 0.01	-	1.92 ± 0.03	-
	2.00	3.94 ± 0.02	100.25 ± 0.01 *RSD = 0.024	3.93 ± 0.02	100.26 ± 0.01 *RSD = 0.024
	5.00	6.92 ± 0.03	99.86 ± 0.04 *RSD = 0.098	6.93 ± 0.01	100.14 ± 0.02 *RSD = 0.049
	7.00	8.94 ± 0.02	100.11 ± 0.05 *RSD = 0.122	8.91 ± 0.03	99.89 ± 0.01 *RSD = 0.025

*Recovery = Found BRO, ng L^− 1^/Real BRO, ng L^− 1^; *RSD Relative standard deviation

#### Stability, selectivity, repeatability, and reproducibility of MIP/S-rGO@NiS_2_NPs/SPR

Stability evaluation of only one MIP/S-rGO@NiS_2_NPs/SPR chip was investigated at 4 ℃. The measurements were taken at weeks 1/2/4/6/8. For this purpose, SPR sensorgrams in presence of 10.0 ng L^− 1^ BRO were obtained during 8 weeks and the obtained SPR signals at the end of 8th week was approximately 98.37% of the obtained SPR signals at the end of the first week, confirming the high stability of the proposed sensor.

**Fig. 6:**
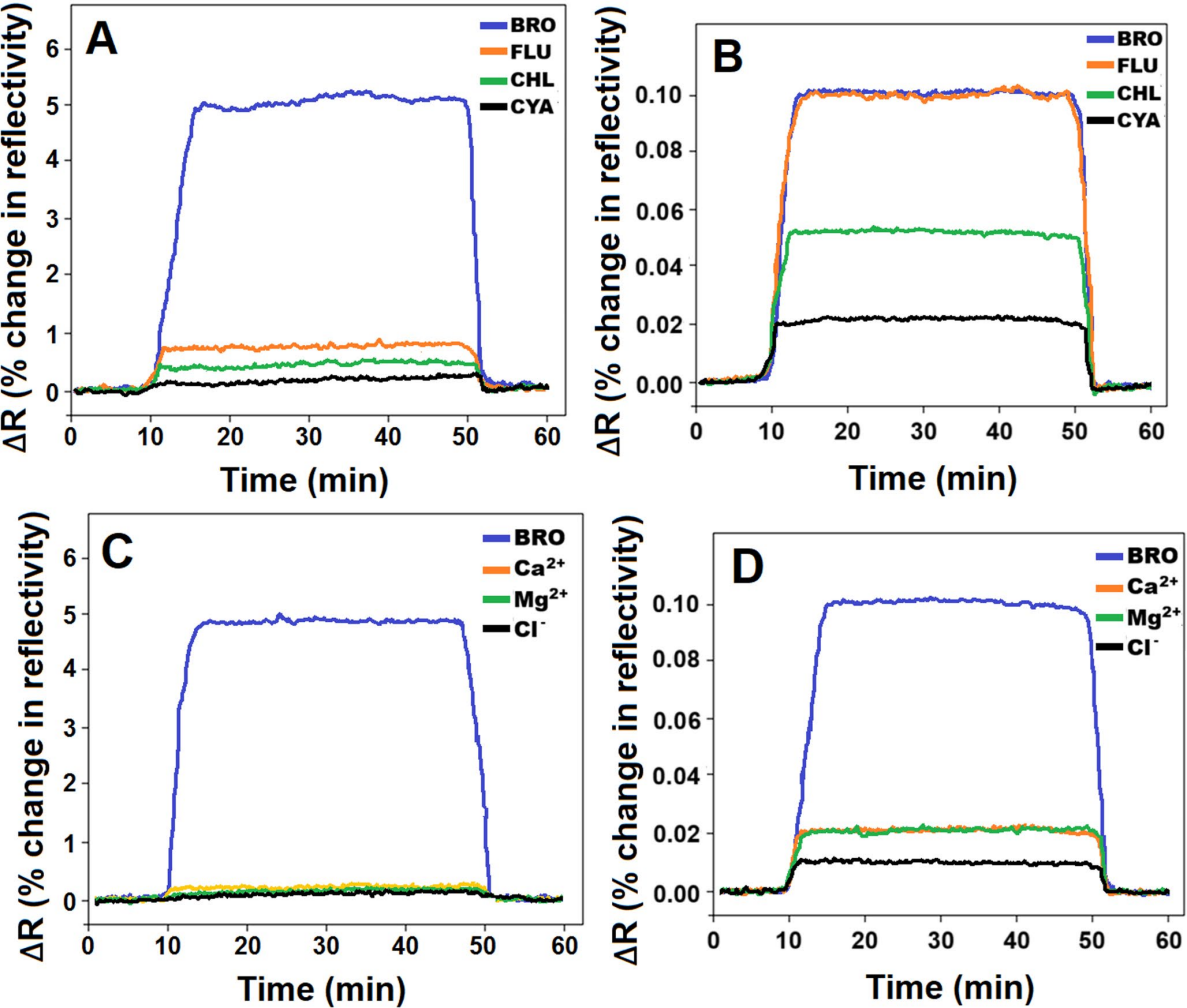
Selectivity studies at (**A**) MIP/S-rGO@NiS_2_NPs/SPR chip, (**B**) NIP/S-rGO@NiS_2_NPs/SPR chip in 10.0 ng L^− 1^ BRO, 1000.0 ng L^− 1^ FLU, 1000.0 ng L^− 1^ CHL, and 1000.0 ng L^− 1^ CYA, (**C**) Selectivity studies at MIP/S-rGO@NiS_2_NPs/SPR chip in 10.0 ng L^− 1^ BRO, 1000.0 ng L^− 1^ Ca^2+^, 1000.0 ng L^− 1^ Mg^2+^, and 1000.0 ng L^− 1^ Cl^−^, (**D**) Selectivity studies at NIP/S-rGO@NiS_2_NPs/SPR chip in 10.0 ng L^− 1^ BRO, 1000.0 ng L^− 1^ Ca^2+^, 1000.0 ng L^− 1^ Mg^2+^, and 1000.0 ng L^− 1^ Cl^−^

MIP/S-rGO@NiS_2_NPs/SPR chip depicted superior selectivity for BRO analyte. This information was verified by the SPR chip interaction with 10.0 ng L^− 1^ BRO and 1000.0 ng L^− 1^ concentrations of FLU, CHL, CYA, common ions including Ca^2+^, Mg^2+^ and Cl^−^ (Fig. 6A and C). FLU, CHL, and CYA were selected as the competing agents, which belong to the class of organic halide pesticides containing diamide [39] and these compounds have great potential for use in integrated pest management strategies [40]. The common ions including Ca^2+^, Mg^2+^ and Cl^−^ were also selected due to the general interferences in water samples. The subsequent calculations of selectivity (k) and relative selectivity (k’) coefficients (Table S1) substantiated these findings, demonstrating that MIP/S-rGO@NiS_2_NPs/SPR chip could detect BRO with increased selectivity, 10.0 times greater than for FLU, 20.0 times greater than for CHL, 50.0 times greater than for CYA, 50.0 times greater than for Ca^2+^, 100.0 times greater than for Mg^2+^ and 166.67 times greater than for Cl^−^. Moreover, Fig. 6B and D highlighted the achieved selectivity through molecularly imprinted technology in comparison with NIP/S-rGO@NiS_2_NPs/SPR chip and k’ values between 16.7 and 20.0 (Table S1) indicated MIP/S-rGO@NiS_2_NPs/SPR chip’s high specificity for BRO detection.

**Fig. 7:**
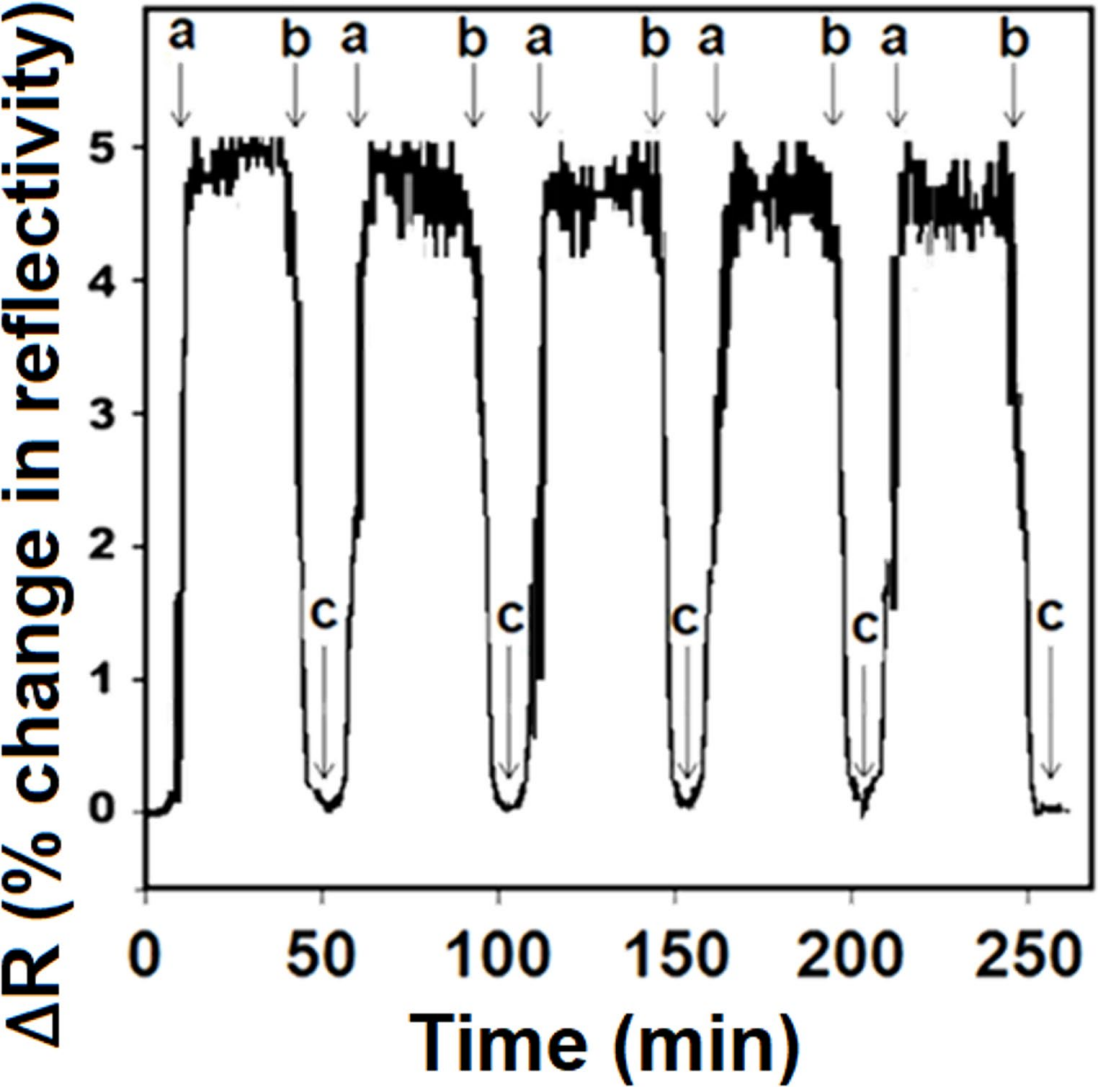
Repeatability of MIP/S-rGO@NiS_2_NPs/SPR chip: (**a**) adsorption; (**b**) desorption; (**c**) regeneration

To evaluate the repeatability of a single MIP/S-rGO@NiS_2_NPs/SPR chip, five successive “adsorption–desorption–regeneration” cycles were performed utilizing 10.0 ng L^− 1^ BRO. The consistent SPR signals of approximately 5.0 ∆R with a relative standard deviation (RSD) of 0.32% were observed along all five cycles (Fig. 7), thus verifying the good repeatability of MIP/S-rGO@NiS_2_NPs/SPR chip.

To reveal the reliability of the sample preparation procedure and assess reproducibility, 15 independent MIP/S-rGO@NiS_2_NPs/SPR chips were fabricated at same batches via the procedures described in Sect. 2.4 and subsequently interacted with 10.0 ng L^− 1^ BRO. The resulting SPR signals from these 15 independent SPR chips produced a RSD of 0.49%, which indicating the high level of reproducibility of the sample preparation method.

## Conclusion

A novel SPR sensor, which was developed for the precise detection of broflanilide in the bottled drinking water and rice samples, was based on MIPs combined with sulphur-doped reduced graphene oxide@nickel sulfide nanoparticles. This sensor, fabricated through a hydrothermal treatment process, revealed exceptional analytical performance such as remarkable selectivity, sensitivity, and repeatability. It achieved a linear detection range of 1.0–10.0 ng L^− 1^ and a low detection limit of 0.33 ng L^− 1^. In addition, experimental results indicated this SPR sensor’s accuracy with high recoveries, ranging between 99.86% and 100.25%. The successful application of this advanced SPR sensor will provide its significant potential for future applications in detecting a wide array of other insecticides.

## Supplementary Information

Below is the link to the electronic supplementary material.

Supplementary Material 1

## Data Availability

No datasets were generated or analysed during the current study.
