# Supplementary material for: A novel surface plasmon resonance sensor development for broflanilide determination using molecularly imprinted polymers and sulphur doped reduced graphene oxide@nickel sulfide nanoparticles
Source: Mikrochim Acta. 2026 Apr 14;193(5):314. doi: 10.1007/s00604-026-08041-3 (PMC13079531; doi:10.1007/s00604-026-08041-3)
Supplement: Supplementary file 1 — Supplementary Material 1 [file 604_2026_8041_MOESM1_ESM.docx]

**Supplementary Data**

**For**

**A novel surface plasmon resonance sensor development for broflanilide determination using molecularly imprinted polymers and sulphur doped reduced graphene oxide@nickel sulfide nanoparticles**

Mustafa Anıl Erbağcı^1^, Kaan Kaplan^2^, Neslihan Özdemir^3^, Hüseyin Enes Altınok^4^, Mehmet Lütfi Yola^5,6*^

*^1^Department of Nutrition and Dietetics, Faculty of Health Sciences, Hasan Kalyoncu University, Gaziantep, 27010, Türkiye*

*^2^Department of Aerospace Engineering, Sivas Science and Technology University, Sivas, 58000, Türkiye*

*^3^Department of Machinery and Metal Technologies, Merzifon Vocational School, Amasya University, Amasya, 05300, Türkiye*

*^4^Department of Chemical Engineering, Faculty of Engineering, Pamukkale University, Denizli, 20160, Türkiye*

*^5^Department of Biology, Faculty of Science, Ankara University, Ankara, 06100, Türkiye*

*^6^Integrated Technologies Research Center (BUTAM), Ankara University, Ankara, 06690, Türkiye*

**Correspondence: mehmetlutfiyola@ankara.edu.tr; Tel.: +90-3122168600; Fax: +90-3122868900*

**Materials and Instrumentation**

Scanning electron microscopy (SEM, ZEISS EVO 50 SEM, Tokyo, Japan), Fourier Transform Infrared Spectroscopy (FTIR, Bruker Optics Inc., Ettlingen, Germany), and Rigaku X-ray diffractometer (XRD, Germany) were used for the structural characterizations. Nano magnetics instrument mode atomic force microscopy (AFM, Tokyo, Japan) was used for the observation of surface thicknesses. Ellipsometer measurements were conducted by using an auto-nulling imaging ellipsometer (Nanofilm EP3, Germany) and the measurements were carried out at six different points of the SPR chip. SPR system (GenOptics, SPRi-Lab, Orsay, France) was used for analytical applications.

**Sensitivity of MIP film modified S-rGO@NiS_2_NPs-functionalized SPR chip**

*LOQ = 10.0 S / m*

*LOD = 3.3 S / m*

S: Standard deviation of the intercept and m*:* Slope of the regression line

**Fig. S1** (A) EDX image of S-rGO@NiS_2_NPs nanocomposite and (B) N_2_ adsorption-desorption isotherms of rGO, S-rGO, NiS_2_NPs, and S-rGO@NiS_2_NPs nanocomposite


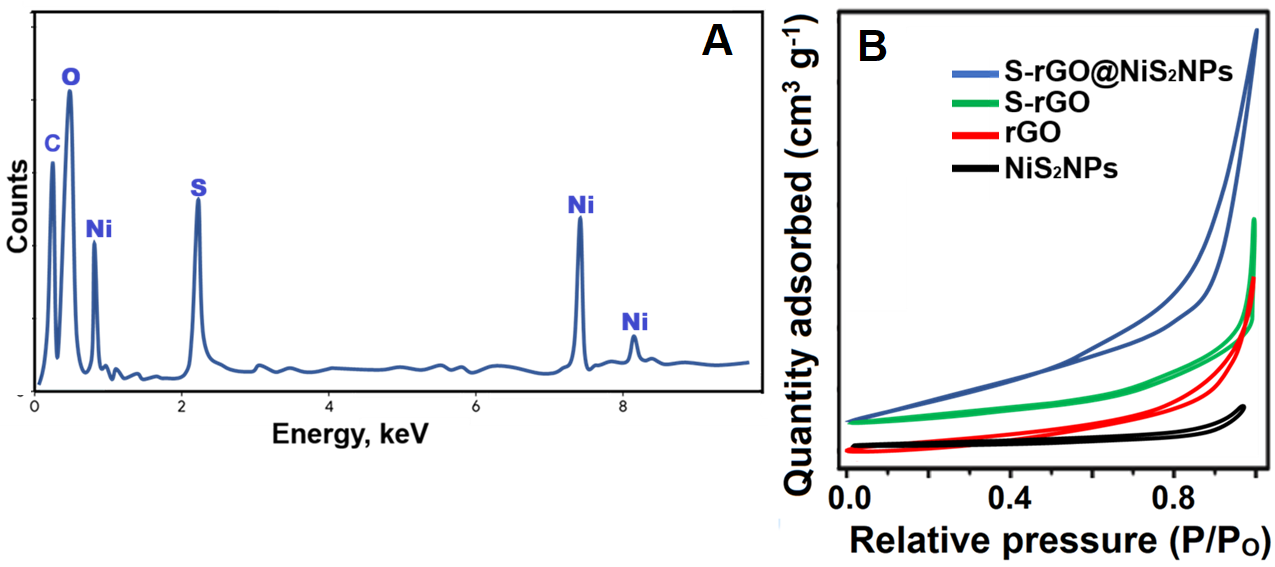

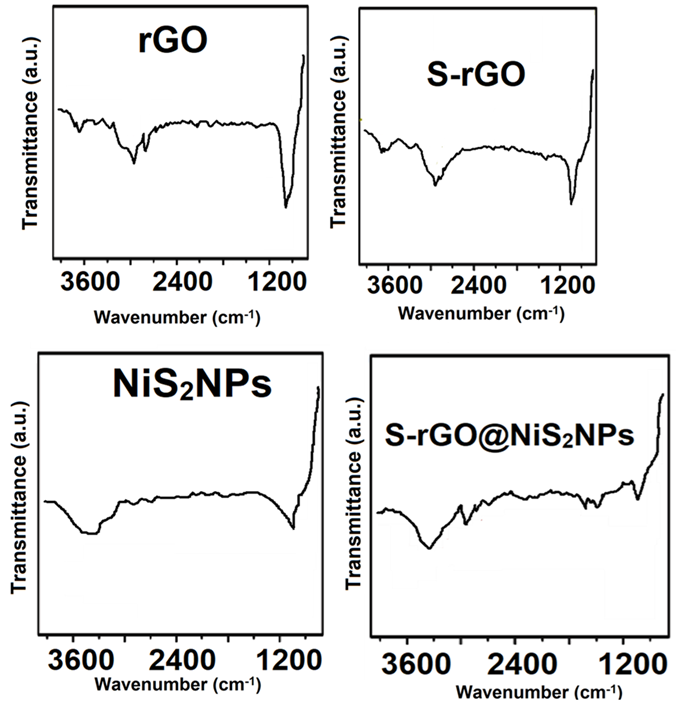


**Fig. S2** FTIR spectra of rGO, S-rGO, NiS_2_NPs, and S-rGO@NiS_2_NPs nanocomposite

**Fig. S3** C1s XPS spectra of (A) rGO and (B) S-rGO; (C) S2p XPS spectra of S-rGO


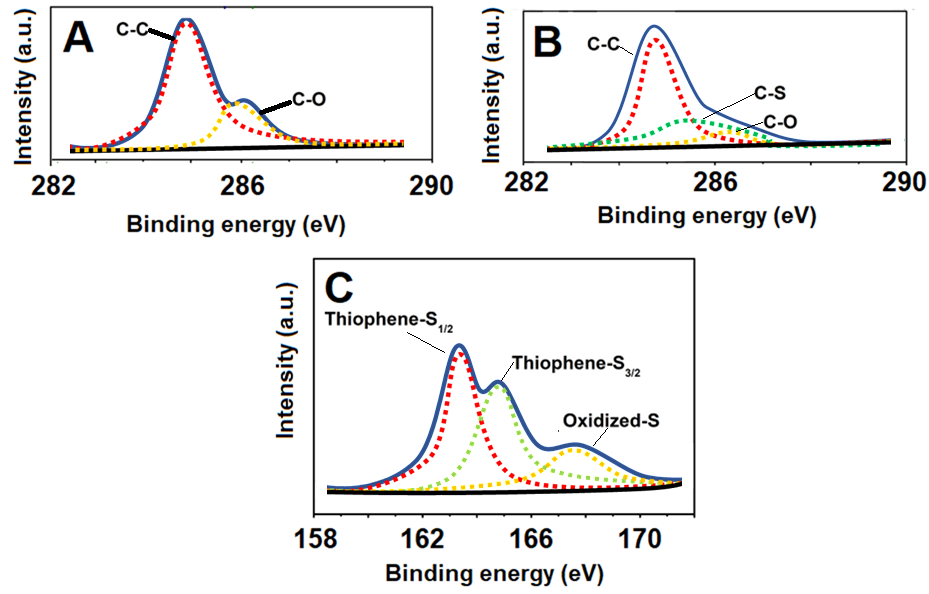


**Fig S4** Ellipsometry image of BRO-imprinted film on S-rGO@NiS_2_NPs/SPR surface


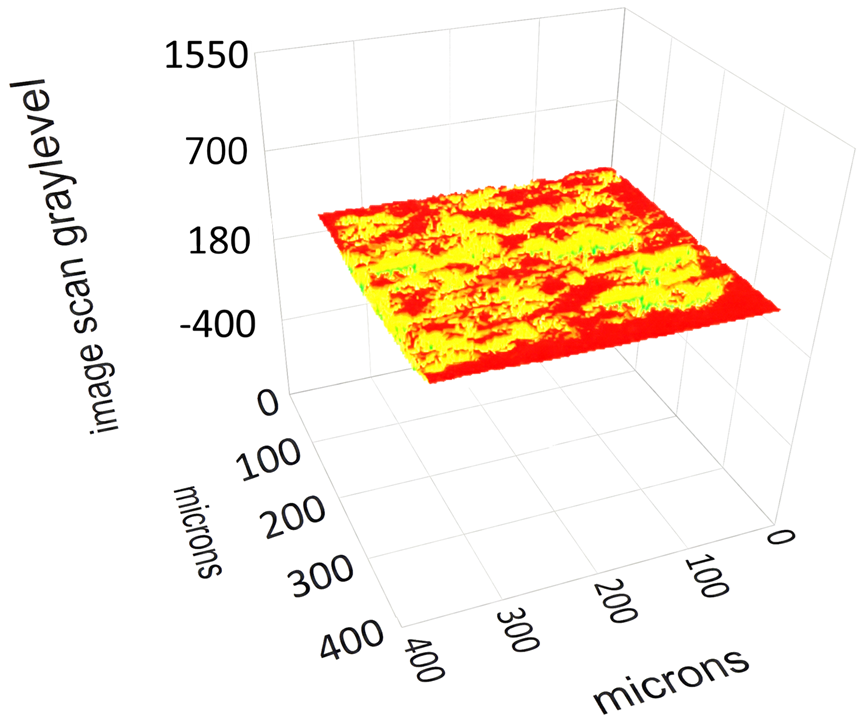


**Fig. S5** Effect of (A) mole ratio and (B) desorption time on SPR signals in presence of 10.0 ng L^-1^ BRO


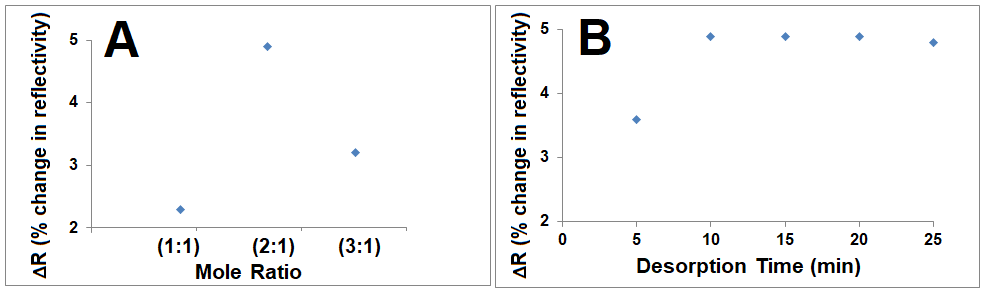


**Table S1.** k and k′ values of MIP/S-rGO@NiS_2_NPs/SPR chip and NIP/S-rGO@NiS_2_NPs/SPR chip (*n*=6)

|  | **MIP** | | **NIP** | |  |
| --- | --- | --- | --- | --- | --- |
|  | **∆R** | **k** | **∆R** | **k** | **k′** |
| BRO | 5.00 ± 0.01 | - | 0.10 ± 0.02 | - | - |
| FLU | 0.50 ± 0.01 | 10.0 | 0.10 ± 0.01 | 1.00 | 10.0 |
| CHL | 0.25 ± 0.02 | 20.0 | 0.05 ± 0.01 | 2.00 | 10.0 |
| CYA | 0.10 ± 0.05 | 50.00 | 0.02 ± 0.04 | 5.00 | 10.0 |
| Ca^2+^ | 0.10 ± 0.02 | 50.00 | 0.02 ± 0.02 | 5.00 | 10.0 |
| Mg^2+^ | 0.05 ± 0.03 | 100.00 | 0.02 ± 0.01 | 5.00 | 20.0 |
| Cl^-^ | 0.03 ± 0.01 | 166.67 | 0.01 ± 0.01 | 10.00 | 16.7 |

Analyte concentrations: 10.0 ng L^-1^ BRO, 1000.0 ng L^-1^ FLU, 1000.0 ng L^-1^ CHL, 1000.0 ng L^-1^ CYA, 1000.0 ng L^-1^ Ca^2+^, 1000.0 ng L^-1^ Mg^2+^, and 1000.0 ng L^-1^ Cl^-^
